# Exploring the influence of digital parental awareness on digital play addiction among preschoolers

**DOI:** 10.3389/fpsyg.2025.1730555

**Published:** 2026-01-09

**Authors:** Yüksel B. Yüksel Aykanat, Büşra Somuncu Çoksağır, Alanoud A. Albatli, Selahattin Semiz, Juan Gómez-Salgado, Murat Yıldırım

**Affiliations:** 1 Department of Early Childhood Education, Erciyes University, Kayseri, Türkiye; 2 Department of Early Childhood Education, Anadolu University, Eskişehir, Türkiye; 3 Early Childhood Education Department, College of Education, Imam Mohammad Ibn Saud Islamic University, Riyadh, Saudi Arabia; 4 Department of Early Childhood Education, Agri Ibrahim Cecen University, Ağrı, Türkiye; 5 Department of Sociology, Social Work and Public Health, Faculty of Labour Sciences, University of Huelva, Huelva, Spain; 6 Safety and Health Postgraduate Program, Universidad Espíritu Santo, Guayaquil, Ecuador; 7 Department of Psychology, Faculty of Science and Letters, Agri Ibrahim Cecen University, Ağrı, Türkiye; 8 Psychology Research Centre, Khazar University, Baku, Azerbaijan

**Keywords:** addiction, digital parenting, digital play, early childhood, structural equation modeling

## Abstract

**Background:**

The widespread use of digital devices has made digital play an important part of children’s daily lives. While digital play provides entertainment and engagement, excessive use may lead to addiction-like behaviors, prompting concerns among parents about their children’s digital wellbeing. This shift has redefined parenting roles, emphasizing the importance of digital parenting awareness.

**Objective:**

This study aimed to investigate the relationship between parents’ digital awareness and their children’s tendencies toward digital play addiction in early childhood.

**Methods:**

A cross-sectional design was employed, utilising structural equation modeling. The study was conducted in Türkiye with a sample of 673 parents of children aged 4–6 years enrolled in state preschools. Data were collected through the Personal Information Form, the Digital Parenting Awareness Scale, and the Digital Play Addiction Tendency Scale.

**Results:**

Structural equation modeling revealed that digital negligence and being a negative role model significantly predicted all dimensions of children’s digital play addiction tendency, namely dissociation from life, conflict, constant play, and reflection on life. In contrast, efficient use showed no significant effect. Protective behaviors aimed at minimizing digital risks were significant negative predictors across all addiction dimensions.

**Conclusion:**

Parental behaviors play a critical role in influencing children’s digital play habits. While neglect and negative role modeling increase addiction tendencies, protective digital parenting practices serve as effective buffers. These findings suggest the need for targeted parental guidance and digital literacy interventions in early childhood settings.

## Introduction

In today’s world, the challenges of parenting extend beyond the physical environment into the digital realm. The rapid advancement of digital technologies has redefined traditional parenting roles, necessitating a shift toward “digital parenting” to ensure children’s safe and responsible use of technology (Benedetto and Ingrassia, 2021; Choy et al., 2024). While parents often view digital tools as opportunities for learning, children frequently engage with these devices primarily for gaming (Arnott and Yelland, 2020; Işıkoğlu Erdoğan, 2019). Although digital play is a normative part of modern childhood (Scott et al., 2023), the immersive nature of modern games and the lack of self-regulation mechanisms in young children can lead to toward problematic digital play, specifically digital play addiction tendencies. This aligns with broader clinical perspectives suggesting that behavioral addictions tendencies often stem from deficits in impulse control, self-regulation, and compulsive reward-seeking mechanisms (Raffone et al., 2025). Furthermore, research on neurodevelopmental conditions highlights that deficits in executive functioning and emotional regulation are core mechanisms underlying various compulsive behaviors and comorbidities, reinforcing the link between dysregulation and addiction vulnerability (Barlattani et al., 2023). Unlike high engagement, digital play addiction tendencies arecharacterized by impaired control and the prioritization of gaming over daily activities.

**Figure 1:**
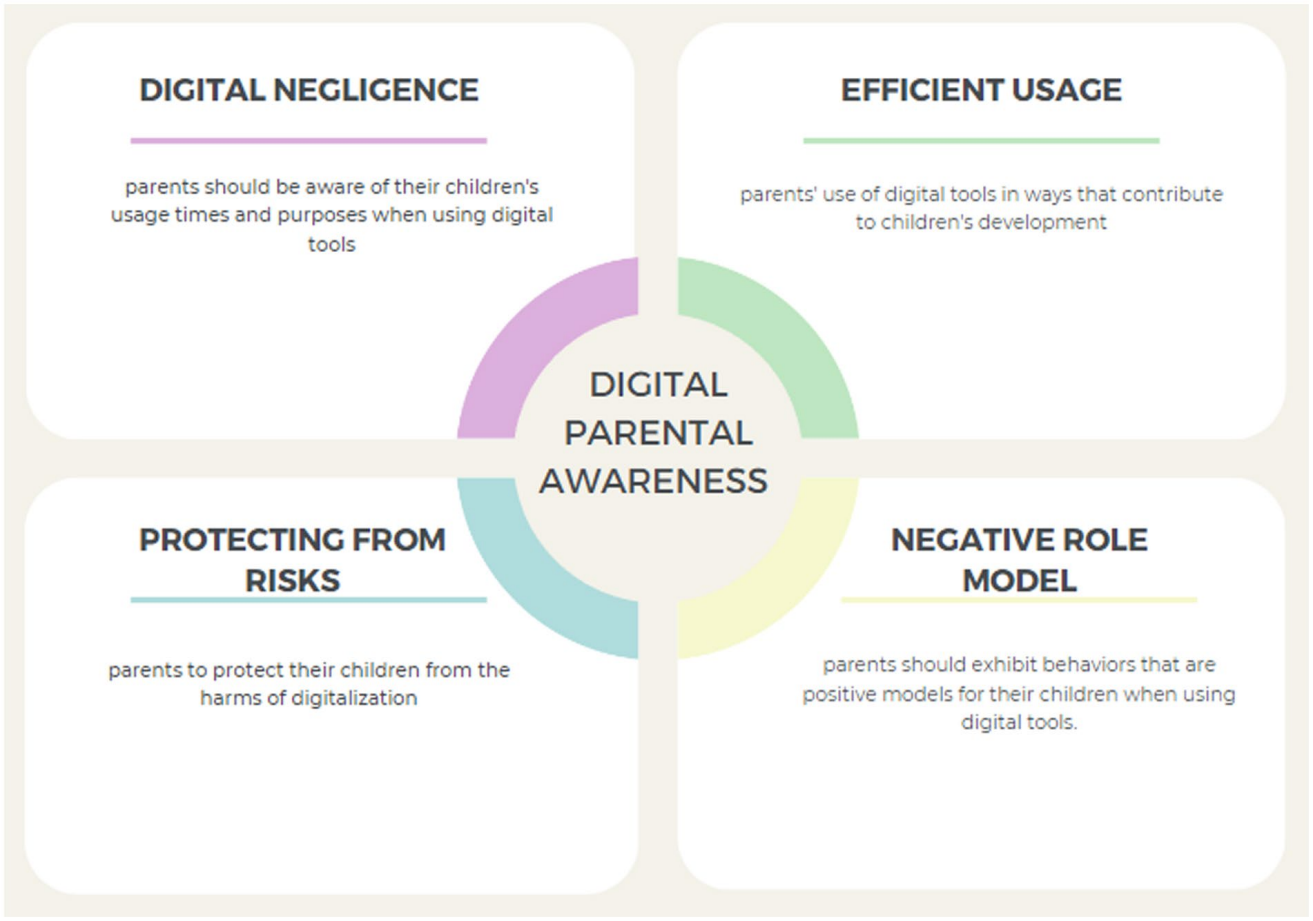
Subdimensions of digital parent awareness (Figure created by the authors, adapted from Manap and Durmuş, 2020).

Despite the critical role of parents, there is a notable limitation in the existing literature. Previous studies have predominantly focused on general online risks (Livingstone et al., 2017) or broad parental mediation strategies (Gözüm and Kandır, 2021; Toran et al., 2024). While existing research highlights concerns about digital media’s role in children’s lives (Stoilova et al., 2021; Wolfers et al., 2024), there remains a distinct gap regarding how specific dimensions of parental digital awareness directly shape digital play addiction tendencies in early childhood. These specific dimensions—namely digital negligence, negative role modeling, efficient use, and risk protection—constitute the conceptual framework of parental digital awareness visualized in Figure 1. Unlike mediation strategies, which describe what parents do, or digital literacy, which captures technical skill, digital awareness reflects a more reflective and meaning-making process that may underlie parental choices. Addressing this gap is crucial, as understanding the relationship between this multifaceted awareness framework and children’s addiction tendencies can inform more effective strategies to place children’s relationships with digital games on healthier ground (Livingstone and Byrne, 2018; Yurdakul et al., 2013).

### Present study

To address the identified gap regarding the role of parental awareness, this research utilizes the Ecological Systems Theory (Bronfenbrenner, 2005). This theory posits that child development is shaped by the reciprocal interactions between the individual and their multi-layered environment. Among these layers, the microsystem—which includes parents, teachers, and peers—is the most influential context for early development. Within this system, parents act as the primary mediators, regulating the child’s environment and experiences.

**Figure 2:**
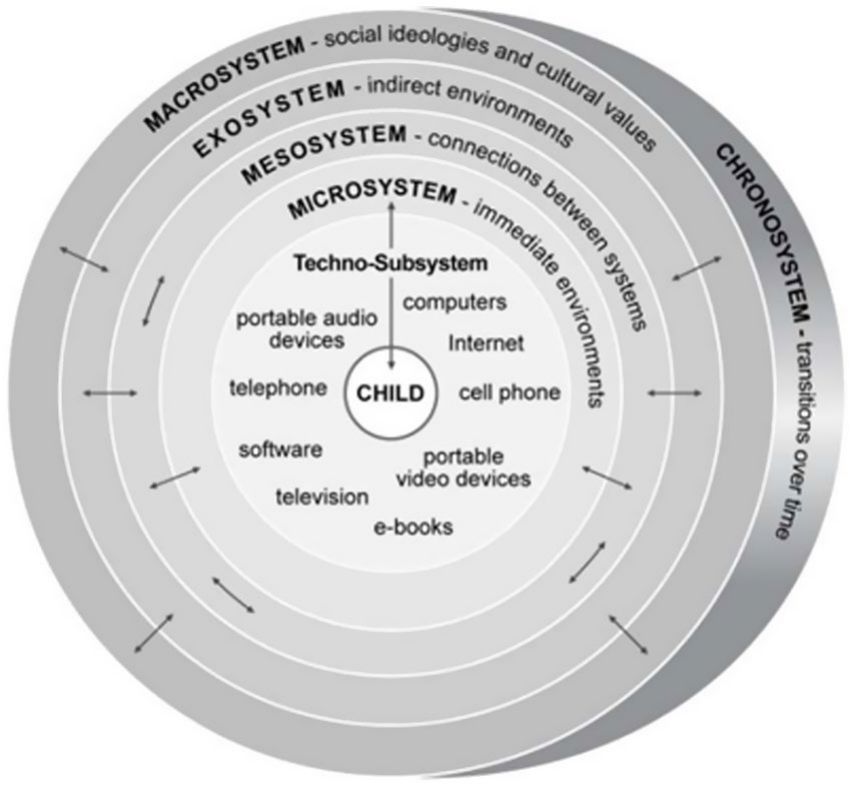
The ecological techno-subsystem (Figure created by the authors, adapted from Johnson and Puplampu, 2008).

However, the pervasive nature of modern technology necessitates an expansion of this traditional framework. Johnson and Puplampu (2008) introduced the concept of the “techno-subsystem” to describe the child’s direct interaction with digital technologies within the microsystem. As digital tools become integral to the child’s ecosystem, the interaction becomes more complex, bidirectional, and dynamic. In this context, parents are not just passive observers but active regulators of this techno-subsystem. As illustrated in Figure 2, parental digital awareness acts as a filter that shapes the quality of the child’s interaction with the digital world.

Grounded in this theoretical model, the present study argues that families must possess specific “digital parental awareness” skills—including preventing negligence, avoiding negative modeling, ensuring efficient use, and protecting from risks—to manage this techno-subsystem effectively. Conceptually, this form of parental awareness mirrors psychological processes of metacognitive monitoring and affective attunement, which are essential for maintaining presence and connection in interpersonal dynamics (Sarasso et al., 2024). We hypothesize that deficiencies in these specific parental skills directly contribute to digital play addiction tendencies in young children.

Accordingly, to investigate the impact of parents’ digital awareness on young children’s tendencies toward digital play addiction, the following hypotheses were tested:

1. Parents’ digital negligence influences children’s digital play addiction tendencies.
2. Parents’ negative role-modeling behaviors influence children’s digital play addiction tendencies.
3. Parents’ efficient use of digital tools influences children’s digital play addiction tendencies.
4. Parents’ protective behaviors against digital risks influence children’s digital play addiction tendencies.

By testing these hypotheses, this study aims to clarify how specific parental digital behaviors shape children’s engagement with digital play. Understanding these dynamics is essential for developing evidence-based strategies that preserve the developmental value of play while minimizing addiction risks in early childhood.

## Method

### Research design

This study adopted a descriptive and relational design within the framework of quantitative research methods to explore the relationship between young children’s digital play addiction tendencies and parents’ awareness of digital parenting. A survey methodology was employed to collect data. The research was conducted using a cross-sectional design during the spring semester of 2022. Convenience sampling was employed in the study. The data for this study were collected online from participants residing in two different cities in Turkey. The researchers visited state kindergartens and distributed the link to the online survey (hosted on Google Forms) to parents via classroom teachers.

### Participants

The sample consisted of 673 parents residing in Türkiye, each of whom had a child aged between 4 and 6 years enrolled in a state preschool. The mothers’ ages ranged from 23 to 54 years (*M* = 33), while the fathers’ ages ranged from 22 to 59 years (*M* = 36). Regarding educational background, 21.1% of the mothers had completed primary school, 37.6% had completed secondary school or high school, 39.4% had attended college or university, and 1.9% held a master’s or doctoral degree. Among fathers, 14.6% had completed primary school, 37.6% secondary school or high school, 43.6% college or university, and 4.3% held master’s or doctoral degrees. In terms of socioeconomic status, 80.83% of the families were in the low socioeconomic level group, 13.67% in the middle, and 5.50% in the high socioeconomic level group. Of the children represented in the sample, 52.9% were male (*n* = 356) and 47.1% were female (*n* = 317). Regarding age distribution, 31.4% of the children were 4 years old (*n* = 211), 32.2% were 5 years old (*n* = 217), and 36.4% were 6 years old (*n* = 245) (Table 1).

**Table 1:** Demographics of the participants.

Variable	Group	*n*	%
Child gender	Male	356	52.9
	Female	317	47.1
Child age	4	211	31.4
	5	217	32.2
	6	245	36.4

### Measures

#### Digital Parental Awareness Scale

Manap and Durmuş (2020) developed the Digital Parental Awareness Scale to measure how aware parents are of their digital parenting practices. The scale includes four dimensions: digital negligence (DN), being a negative role model (NRM), efficient use (EU), and protection from risks (PFR). It contains 16 items rated on a 5-point Likert scale, ranging from 1 (never) to 5 (always). Higher scores on each subscale indicate a greater presence of the respective behavior. The internal consistency coefficients for the subscales were as follows: digital negligence *α* = 0.843, negative role modeling *α* = 0.784, efficient use *α* = 0.799, and protection from risks *α* = 0.707.

#### Digital Play Addiction Tendency Scale

Budak and Işıkoğlu (2022) created the Digital Play Addiction Tendency Scale to assess the extent to which young children show signs of digital play addiction. The scale evaluates four key dimensions: dissociation from life (DFL), conflict (CT), constant play (CP), and reflection on life (RL). It consists of 20 items, each rated on a 5-point Likert scale from 1 (never) to 5 (always). Higher scores reflect stronger addiction-like tendencies in each domain. The internal consistency coefficients for the subscales were: dissociation from life *α* = 0.938, conflict *α* = 0.931, constant play *α* = 0.917, and reflection on life *α* = 0.815.

#### Procedure

Before data collection, ethical approval was obtained from the Ethics Committee of Ağrı İbrahim Çeçen University. Participants were fully informed about the purpose, scope, and voluntary nature of the study. Data were collected through self-report online surveys administered to parents of children aged 4–6 years enrolled in state preschools across Türkiye. All participants provided informed consent before taking part in the study.

### Data analysis

Descriptive analysis was conducted using SPSS version 26, while SmartPLS 4 software was used for structural equation modeling. The structural equation model can analyze multiple variables simultaneously while considering measurement errors through latent variables, thus enhancing the study’s validity and reliability. When analyzing complex and causal relationships, using variance-based methods like PLS rather than covariance-based methods is advisable (Hair et al., 2022; Hair et al., 2011). Although the sample size was adequate for covariance-based SEM, PLS-SEM was selected because the main objective of this research was to maximize the explained variance and evaluate predictive capability rather than to validate an existing theory. Moreover, PLS-SEM handles non-normal data and complex measurement structures more effectively, making it the most appropriate method for this study (Hair et al., 2022). PLS path modeling was used to create the model, which was then evaluated for convergent and discriminant validity in the measurement model. Subsequently, the structural model was assessed using bootstrapping analysis.

## Results

### Measurement model assessment

During the evaluation of the measurement model, the convergent validity was assessed by checking Cronbach’s alpha, item loadings, CR, and AVE values. Cross loadings results were evaluated based on recommended loading thresholds, where values ≥ 0.70 are considered ideal, loadings between 0.60 and 0.70 acceptable, loadings between 0.40 and 0.60 marginal but retainable with theoretical justification, and loadings <0.40 unacceptable (Hair et al., 2022). As depicted in Table 2, the loadings of the items range from 0.572 to 0.930. Among all items, only item PFR2 fell below the 0.60 threshold. However, given that this item represents a theoretically important component of the corresponding factor, its loading is very close to the 0.60 cutoff, and the overall scale statistics demonstrate strong psychometric properties, retaining the item was deemed appropriate. Based on the results of Cronbach’s alpha and composite reliability (CR), all values exceeded 0.7. Moreover, it is worth noting that the AVE values are all above 0.5, ranging from 0.517 to 0.785. After examining the VIF values of the items, it was found that all values met the assumption of multicollinearity (O’brien, 2007). From the information presented, it is clear that there is strong evidence for establishing convergent validity (Hair et al., 2022).

**Table 2:** Convergent validity.

Variable	Item	Loading	VIF	*α*	CR	AVE
CP	CP1	0.783	1.923	0.917	0.938	0.752
	CP2	0.872	2.740			
	CP3	0.881	2.922			
	CP4	0.898	3.333			
	CP5	0.897	3.164			
CT	CT1	0.869	2.806	0.931	0.948	0.785
	CT2	0.912	4.069			
	CT3	0.930	4.858			
	CT4	0.868	2.779			
	CT5	0.850	2.549			
DFL	DFL1	0.780	2.188	0.938	0.950	0.731
	DFL2	0.836	2.536			
	DFL3	0.884	3.729			
	DFL4	0.884	3.517			
	DFL5	0.874	3.327			
	DFL6	0.829	2.476			
	DFL7	0.890	3.607			
DN	DN1	0.767	1.585	0.843	0.895	0.680
	DN2	0.858	2.033			
	DN3	0.813	1.867			
	DN4	0.857	2.152			
EU	EU1	0.815	1.583	0.799	0.863	0.614
	EU2	0.826	1.762			
	EU3	0.624	1.426			
	EU4	0.848	1.843			
NRM	NRM1	0.709	1.379	0.784	0.861	0.608
	NRM2	0.764	1.502			
	NRM3	0.820	1.727			
	NRM4	0.821	1.765			
PFR	PFR1	0.776	1.304	0.707	0.808	0.517
	PFR2	0.572	1.315			
	PFR3	0.817	1.377			
	PFR4	0.688	1.376			
RL	RL1	0.845	1.739	0.815	0.891	0.731
	RL2	0.887	2.096			
	RL3	0.831	1.738			

DN, digital negligence; NRM, being a negative role model; EU, efficient use; PFR, protection from risks; DFL, dissociation from life; CT, conflict; CP, constant play; RL reflection on life.

We employed the Fornell–Larcker criterion and the HTMT ratio to evaluate the data in assessing discriminant validity. Based on the analysis presented in Tables 3, 4, we can conclude that both the Fornell–Larcker criterion (which indicates that the square root values of the Average Variance Extracted (AVE) of all constructs are more significant than their respective inter-structure correlations) and the HTMT ratio criterion (which requires that all values be lower than 0.9) have been met (Şahin et al., 2022). Thus, discriminant validity was established (Fornell and Larcker, 1981; Hair et al., 2022). SmartPLS generates only the SRMR (standardized root mean square residual) value from the model fit indices. The SRMR value for the model is 0.049.

**Table 3:** HTMT ratio (discriminant validity).

Variable	CP	CT	DFL	DN	EU	NRM	PRF	RL
CP								
CT	0.885							
DFL	0.830	0.859						
DN	0.597	0.572	0.566					
EU	0.247	0.264	0.285	0.176				
NRM	0.506	0.486	0.517	0.828	0.199			
PRF	0.270	0.316	0.332	0.182	0.891	0.159		
RL	0.744	0.786	0.839	0.487	0.141	0.461	0.199	

DN, digital negligence; NRM, being a negative role model; EU, efficient use; PFR, protection from risks; DFL, dissociation from life; CT, conflict; CP, constant play; RL, reflection on life.

**Table 4:** Fornell–Larcker criterion (discriminant validity).

Variable	CP	CT	DFL	DN	EU	NRM	PRF	RL
CP	0.867							
CT	0.839	0.886						
DFL	0.772	0.804	0.855					
DN	0.526	0.510	0.507	0.825				
EU	−0.235	−0.249	−0.274	−0.164	0.784			
NRM	0.433	0.418	0.446	0.678	−0.168	0.780		
PRF	−0.245	−0.283	−0.300	−0.165	0.714	−0.115	0.719	
RL	0.645	0.687	0.734	0.406	−0.128	0.372	−0.171	0.855

DN, digital negligence; NRM, being a negative role model; EU, efficient use; PFR, protection from risks; DFL, dissociation from life; CT, conflict; CP, constant play; RL, reflection on life.

### Structural model assessment

**Figure 3:**
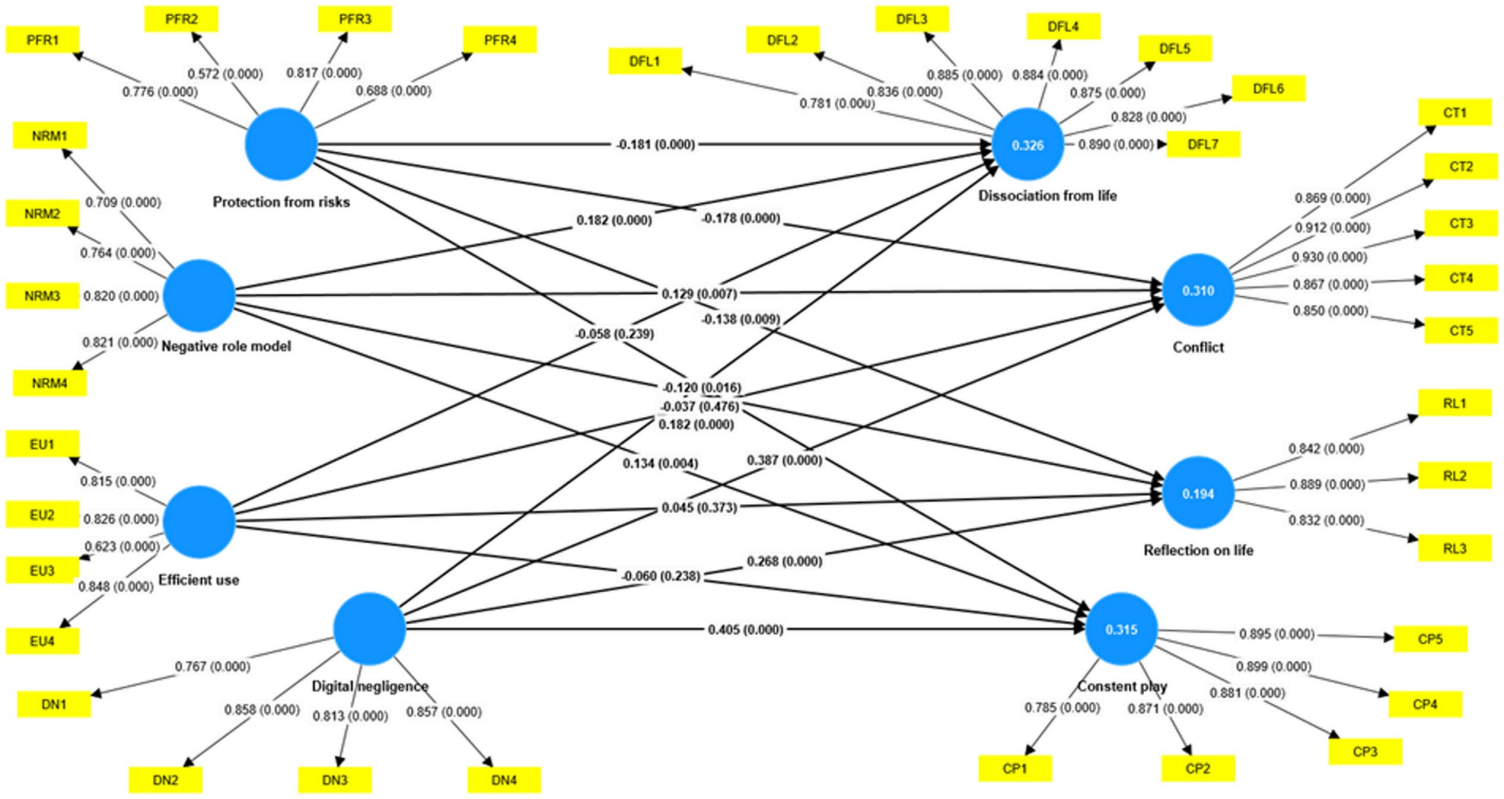
PLS-SEM results.

In this study, the dependent variable was children’s tendency toward digital play addiction, which comprised four factors: dissociation from life (DFL), conflict (CT), constant play (CP), and reflection on life (RL). Parents’ digital parenting awareness comprises four predictor variables: digital negligence (DN), negative role modeling (NRM), efficient use (EU), and protection from risks (PFR). The outcomes of the structural equation modeling have been illustrated in Figure 3 and Table 5.

**Table 5:** Structural modeling results.

Path	Coefficient	VIF	*t* value	*p*-value	Result
DN → CP	0.405	1.880	8.778	0.000	Significant
DN → CT	0.386	1.880	7.804	0.000	Significant
DN → DFL	0.344	1.880	7.344	0.000	Significant
DN → RL	0.267	1.880	5.103	0.000	Significant
EU → CP	−0.060	2.071	1.193	0.233	Not significant
EU → CT	−0.037	2.071	0.728	0.467	Not significant
EU → DFL	−0.058	2.071	1.216	0.224	Not significant
EU → RL	0.044	2.071	0.879	0.380	Not significant
NRM → CP	0.135	1.874	2.930	0.003	Significant
NRM → CT	0.129	1.874	2.721	0.007	Significant
NRM → DFL	0.183	1.874	4.006	0.000	Significant
NRM → RL	0.182	1.874	3.718	0.000	Significant
PRF → CP	−0.120	2.059	2.395	0.017	Significant
PRF → CT	−0.178	2.059	3.566	0.000	Significant
PRF → DFL	−0.180	2.059	3.568	0.000	Significant
PRF → RL	−0.138	2.059	2.627	0.009	Significant

DN, digital negligence; NRM, being a negative role model; EU, efficient use; PFR, protection from risks; DFL, dissociation from life; CT, conflict, CP, constant play; RL, reflection on life.

The study’s findings indicate that children who display signs of digital play addiction, such as dissociation from life (DFL), conflict (CT), constant play (CP), and reflection on life (RL), can be positively influenced by their parents’ actions. Specifically, when parents exhibit digital neglect behaviors (DN), such as ignoring their children’s digital activities or failing to set boundaries, their children are more likely to develop these negative tendencies.

The findings indicate that parents’ behaviors aimed at the efficient use of digital tools do not significantly predict any dimensions of digital play addiction, including dissociation from life (DFL), conflict (CT), constant play (CP), and reflection on life (RL).

In contrast, the study reveals that negative parental behaviors in digital contexts, such as neglect or poor role modeling, are associated with increased risks of digital play addiction in children. These behaviors contribute to heightened levels of dissociation from life (DFL), conflict (CT), constant play (CP), and negative reflection on life (RL).

Moreover, protective parental behaviors (PFR) aimed at minimizing digital risks were found to be significant negative predictors of all dimensions of digital play addiction.

It has been observed that parents’ awareness of digital parenting, which includes digital neglect (DN), efficient use (EU), negative role modeling (NRM), and protection from risks (PFR), is responsible for 33% of the variance in dissociation from life (DFL), 31% of the variance in conflict (CT), 32% of the variance in constant play (CP), and 19% of the variance in reflecting on life (RL). These factors are all sub-factors of digital play addiction tendencies in children. Hence, it is apparent that a parent’s digital awareness is a significant variable that needs to be considered when examining children’s tendencies toward digital play addiction.

## Discussion

Significant findings were obtained in this study regarding the effect of parents’ digital awareness on young children’s digital play addiction tendencies. The first key finding indicates that parents’ digital neglect behaviors significantly increase young children’s digital play addiction tendencies. This relationship can be explained through the “digital nanny” phenomenon, where parents, often due to domestic responsibilities or their own digital engagement (e.g., phubbing, social media use), utilize devices as tools to occupy their children (Manap and Durmuş, 2021; Toran et al., 2016). However, this relationship must be interpreted within the specific socio-cultural dynamics of the Turkish sample rather than as simple parental oversight. National statistics reveal that women in Turkiye devote over four times as much time to household and care activities compared to men (Turkish Statistical Institute and UN Women Turkiye, 2024). In this context, where traditional gender roles often place heavy domestic burdens on mothers, digital devices may serve as a momentary relief mechanism. Yet when parents withdraw their attention in favor of screens, they inadvertently validate the device as a primary source of emotional regulation and entertainment for the child. This lack of parental supervision and interaction creates a cycle where children, deprived of alternative stimuli, become increasingly dependent on the immersive and rewarding nature of digital games (Mercan-Uzun et al., 2023; Toran et al., 2024). This pattern suggests that the results are likely generalizable to other societies with similar collectivist family structures and gendered labor divisions. Thus, digital neglect in this study represents not just a behavioral deficit, but a byproduct of cultural time-poverty that pushes children toward solitary, unregulated digital consumption.

The second finding demonstrates that parents’ negative role modeling significantly predicts children’s addiction tendencies. Children in the early childhood period are in a critical stage of social learning and imitation (Bandura, 1977). When children observe their parents constantly engaging with devices, they internalize this behavior as normative. High parental screen time signals to the child that digital engagement is a prioritized daily activity, thereby reinforcing the child’s own desire for digital immersion (Lusted and Joffe, 2018; Wong et al., 2019). Furthermore, when parents are physically present but mentally absorbed in their devices, children’s unmet needs for attention may drive them further into the virtual world as a compensatory mechanism.

Contradictory to some expectations, our study found that parents’ behaviors regarding the “efficient use” of digital tools were not effective in reducing digital play addiction tendencies. While this result contradicts some existing literature (Hidaayah et al., 2020), it implies that ‘efficient use’—often operationalized as selecting educational content or technical management—is insufficient on its own to curb addiction. This non-significant direct relationship suggests that the impact of efficient use is likely mediated by relational factors such as parent–child communication quality and parenting style, which were not explicitly tested in this model. As noted by Livingstone and Byrne (2018), mere access to beneficial content is ineffective without active mediation that scaffolds the child’s experience. If parents enforce ‘efficient’ usage but lack the communicative engagement to discuss and contextualize the content, the digital activity remains a solitary experience for the child. Therefore, the protective potential of efficient use appears to depend not just on *what* content is consumed, but on the *interactional quality* accompanying that consumption.

Finally, the study confirms that parents’ protective behaviors against digital risks are effective in reducing addiction tendencies. Modern parenting involves managing “e-threats,” ranging from inappropriate content to privacy breaches (Tomczyk and Potyrała, 2021). Parents who actively employ security measures—such as parental control software, time restrictions, and content filtering—create a regulated environment that naturally limits the opportunity for addictive behaviors to develop. By controlling the structural parameters of digital play, these parents likely prevent the excessive exposure that leads to dependency (Gözüm and Kandır, 2021).

### Implications

The findings suggest that combatting digital play addiction tendency requires a multi-faceted approach involving both home and school environments. For parents, the priority should be shifting from passive restriction to active engagement. To reduce digital neglect, parents can implement “device-free zones” or times during the day to foster face-to-face interaction. Instead of using devices as “digital nannies,” parents should be encouraged to co-view or co-play with their children, turning digital time into a shared social activity. Additionally, training programs should move beyond simple awareness to teaching specific digital mediation strategies, helping parents distinguish between “efficient use” and “healthy engagement.” Recent research highlights that digital interventions and technological tools can themselves be effective in enhancing psychological outcomes and literacy (Vignapiano et al., 2025). Therefore, future interventions could utilize mobile applications or digital platforms specifically designed to boost parental awareness and monitoring skills.

Crucially, early childhood educators also play a vital role. Teachers are often the first to notice signs of digital dependency, such as attention deficits or lack of interest in physical play. Educators can act as bridges, organizing workshops to improve families’ digital literacy. Schools can design curricula that not only teach children how to use technology but also how to self-regulate and balance screen time with physical activities. By integrating “digital wellbeing” into parent-teacher meetings, educators can help families create consistent rules across home and school settings.

### Limitations

This study has several limitations that should be considered. First and foremost, the cross-sectional design of the study prevents the establishment of causal inferences between parental awareness and children’s addiction tendencies. While we observed significant associations, longitudinal studies are needed to determine the directionality of these relationships over time. Secondly, data were collected exclusively from parents. Future research would benefit from multi-informant designs that include teacher observations or direct assessments of children’s behavior to reduce self-reporting bias. Third, although the sample size is large, it is limited to the Turkish cultural context. Given that parenting styles and digital habits are culturally embedded, cross-cultural studies are necessary to generalize these findings. Additionally, the developmental characteristics of preschool children should be taken into account when interpreting the results. Preschool children have limited executive functions, immature self-regulation skills, and a natural inclination toward repetitive, immersive play, which may resemble constant engagement or difficulty disengaging from an activity. These behaviors can be normative characteristics of early development rather than indicators of addiction-like pathology. Therefore, the tendencies identified in this study should not be interpreted as clinical addiction but rather as early emerging patterns of problematic or excessive engagement. Finally, the rapid evolution of technology, such as the increasing use of VR/AR in gaming, suggests that future studies should include specific variables related to these emerging technologies.

## Conclusion

This study highlights the critical role of parental digital awareness in shaping young children’s digital habits within the Turkish context. The results reveal that parental neglect and negative role modeling are significant risk factors for digital play addiction tendencies, while protective behaviors against risks serve as a protective shield. Interestingly, “efficient use” alone appeared insufficient to curb addiction tendencies, suggesting a need for deeper digital literacy and active mediation. Consequently, interventions should target not just reducing screen time, but improving the quality of parent–child interactions and equipping both parents and educators with the skills to manage the digital ecosystem. By fostering a conscious and regulated digital environment at home and school, the developmental potential of technology can be harnessed while minimizing the risks of addiction tendency. This study makes an essential contribution to understanding the effects of parents’ digital awareness on young children’s digital play addiction tendencies and sheds light on future research.

## Data Availability

The raw data supporting the conclusions of this article will be made available by the authors, without undue reservation.
